# GLP-1 receptor agonists for the treatment of alcohol use disorder

**DOI:** 10.1172/JCI192414

**Published:** 2025-05-01

**Authors:** Gavin N. Petrie, Leah M. Mayo

**Affiliations:** 1 Department of Psychiatry,; 2 Mathison Centre for Mental Health Education and Research, and; 3 Hotchkiss Brain Institute, University of Calgary, Calgary, Alberta, Canada.

## Abstract

Glucagon-like peptide-1 receptor agonists (GLP-1RAs), such as semaglutide, are widely used in the treatment of metabolic disorders, including type 2 diabetes (T2D) and obesity. These medications primarily function by enhancing insulin secretion; however, emerging evidence suggests that the effects extend beyond metabolic regulation. In this issue of the *JCI*, Farokhnia et al. evaluated the effects of GLP-1RAs alongside another T2D treatment, dipeptidyl peptidase-4 inhibitors (DPP-4Is), on alcohol consumption in humans and preclinical models. In humans, GLP1-RAs, but not DPP-4Is, were associated with reductions in alcohol consumption. Similarly, DPP-4 inhibition had no effect on alcohol intake in rodents. These findings invite further exploration of the mechanisms by which GLP-1RAs reduce alcohol consumption and redefine our pharmacotherapy approach to alcohol use disorder (AUD) by opening the possibility for application as an early harm-reduction tool.

## The GLP-1 system in alcohol use disorder

Glucagon-like peptide-1 receptor agonists (GLP-1RAs), such as semaglutide, are widely recognized for their efficacy in treating metabolic disorders, including type 2 diabetes (T2D) and obesity (1). These medications primarily function by enhancing insulin secretion, delaying gastric emptying, and promoting satiety, thereby reducing overall food intake and supporting weight loss. However, emerging evidence suggests that the effects of GLP-1RAs extend beyond metabolic regulation. Recent research indicates that these agents may also influence the consumption of substances other than food, including alcohol. This discovery — bolstered by 2 recent clinical trials demonstrating reductions in alcohol intake following GLP-1RA treatment (2, 3) — has sparked growing interest in repurposing these medications for alcohol use disorder (AUD), a condition with limited pharmacological treatment options. These findings raise important questions about the underlying mechanisms driving this effect and how we can leverage the GLP-1 system to optimize therapies for AUD.

**Figure 1:**
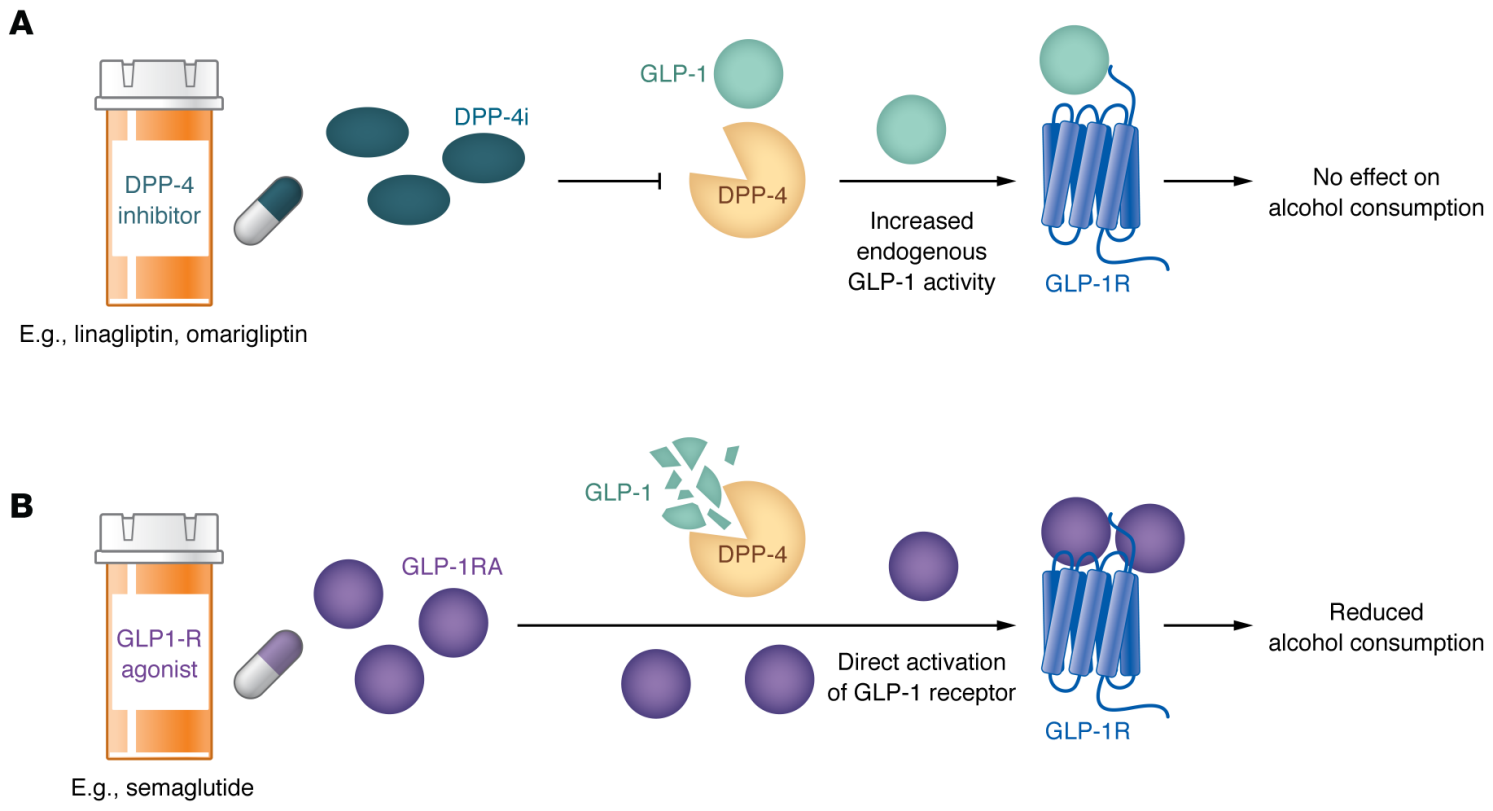
Distinct mechanisms underlie the differential effects of DPP-4Is and GLP-1RAs on alcohol consumption. DPP-4Is (**A**) have no effects on alcohol consumption, while GLP-1RAs (**B**) reduce it. Both drug classes increase activity at the GLP-1 receptor, but through different mechanisms. DPP-4Is, such as linagliptin and omarigliptin, target activity of the DDP-4 enzyme, which normally functions to inactivate GLP-1 and thus prevents it from binding to the GLP-1 receptor. However, inhibition of DDP-4 via an DDP-4I boosts the endogenous GLP-1 signal. Critically, this effect is constrained by the capacity to produce endogenous GLP-1. In contrast, GLP1-RAs, such as semaglutide, are analogs to the GLP-1 peptide that have longer half lives as well as greater potency at, and affinity for, the GLP-1 receptor. The precise mechanisms underpinning the ability of GLP1-RAs to reduce alcohol consumption are, as of yet, unknown, but may relate to these differences in how DPP-4Is and GLP1-RAs affect the GLP-1 system.

In this issue of the *JCI*, Farokhnia and colleagues used a reverse translational approach to examine the effects of enhancing GLP-1R activity — either through synthetic agonists or inhibition of its degrading enzyme, dipeptidyl peptidase-4 (DPP-4) — on alcohol consumption in both humans and preclinical models (Figure 1) (4). In a large cohort study, they analyzed alcohol consumption in patients prescribed GLP-1RAs or DPP-4 inhibitors (DPP-4Is) for metabolic disorders. Only GLP-1RA treatment was associated with reductions in alcohol consumption. These clinical observations were supported by preclinical experiments, in which DPP-4 inhibition had no effect on alcohol intake in rodents. These findings raise important questions about why direct GLP-1R agonism is effective while DPP-4 inhibition is not, highlighting the need to better understand the underlying mechanisms involved in this intervention, and how this knowledge can be leveraged to refine and optimize therapeutic approaches for AUD.

## Contrasting effects of GLP1-RAs and DPP-4Is across species

GLP-1RAs and DPP-4Is both modulate the GLP-1 system but through distinct mechanisms. GLP-1RAs, such as semaglutide and liraglutide, are synthetic analogs resistant to enzymatic degradation, providing prolonged, direct receptor activation. DPP-4Is, like linagliptin and omarigliptin, prevent the rapid breakdown of endogenous GLP-1, modestly extending its half-life. While both enhance glucose-dependent insulin secretion and are used for type 2 diabetes, their effects on alcohol consumption appear to differ substantially.

Farokhnia and colleagues leveraged a unique, real-world electronic health record dataset from the US Department of Veteran Affairs (VA) to gain insight into how GLP-1RAs and DPP-4Is impact alcohol consumption. They focused in on 3 groups: (a) those receiving GLP-1RAs, (b) those receiving DPP-4Is, and (c) those unexposed to either. Critically, alcohol consumption is routinely collected at VA visits via completion of the Alcohol Use Disorders Identification Test–Consumption (AUDIT-C), which is a short tool used to assess alcohol use and frequency. Using propensity matching between the exposure and comparator groups, the authors found that patients exposed to a GLP-1RA had a greater reduction in AUDIT-C scores than unexposed controls, as well as those exposed to DPP-4Is, reflecting a greater reduction in alcohol consumption. In contrast, there was no difference in alcohol consumption between those exposed to DPP-4Is and unexposed controls (4).

From this large cohort study, it appears that GLP1-RAs may reduce alcohol consumption, while DPP-4Is do not (4). Farokhnia and team took it one step further to confirm this hypothesis by leveraging a reverse translational approach (4). The team had already shown that GLP1-RAs can reduce alcohol intake in rats (5). Here, they demonstrated that, as would be predicted, neither DPP-4I linagliptin or omarigliptin were effective in reducing alcohol consumption in rodents (4). Taken together, this series of studies suggests that the relative modest increase of endogenous GLP-1 activity via DPP-4Is may be insufficient to reach a therapeutic threshold. Additionally, because DPP-4 inhibitors rely on endogenous GLP-1, their effects may be further limited by the already rapid degradation and low baseline levels of the peptide, reducing their ability to meaningfully influence neural circuits involved in alcohol consumption. Thus, direct activation of GLP1 receptors via GLP1-RAs may be requisite to leverage this system as a potential therapeutic to reduce alcohol intake.

## A possibility for harm reduction

As research on GLP-1RAs and AUD advances, it is becoming increasingly clear that these medications have the potential to reduce alcohol consumption. However, what sets GLP-1RAs apart from existing pharmacotherapies is not just their efficacy but their potential to shift how we think about AUD treatment. Current AUD pharmacotherapies include naltrexone or acamprosate, both of which work best when patients can abstain prior to the start of treatment, and acamprosate, in particular, is effective in facilitating abstinence-based outcomes, though less effective in treatment models focused on reducing—rather than eliminating—alcohol consumption (6). However, not all patients can, or want to, abstain fully from alcohol use.

GLP-1RAs may be particularly well suited for harm-reduction strategies. Rather than aiming for individuals to commit to complete abstinence, these medications may allow for meaningful reductions in alcohol consumption, decreasing both the intensity and frequency of heavy drinking episodes. A particularly compelling aspect of GLP-1RAs is their effectiveness in individuals who are not actively seeking AUD treatment. Large-scale real-world data and clinical trials indicate that GLP-1RAs reduce alcohol intake even among those without the explicit goal of quitting alcohol consumption (3). This is a critical distinction, as fewer than 10% of individuals with AUD seek treatment, and an even smaller fraction receive pharmacotherapy. By offering a medication that passively reduces alcohol consumption, GLP-1RAs could intervene at earlier stages of problematic drinking, potentially preventing escalation to more severe AUD.

Reduction in alcohol consumption is a particularly relevant outcome given that GLP-1RAs appear to be most effective for individuals with higher baseline alcohol consumption. The current study from Farokhnia and team found that individuals who drink more heavily tended to experience the greatest reductions in alcohol intake when using GLP-1RAs (4). This consideration raises intriguing questions about whether these medications could serve as an early harm-reduction tool for those at risk of progressing to severe AUD, even before they recognize their drinking as problematic. Rather than viewing pharmacotherapy as a last resort for treatment-resistant individuals, GLP-1RAs could normalize the use of medication as a preventative measure — much like statins for cardiovascular disease or metformin for prediabetes.

Another key consideration is the relationship between BMI and treatment response. While GLP-1RAs are most effective in individuals with a BMI over 30, their alcohol-reducing effects are not necessarily tied to body weight. This possibility broadens the potential application of these medications, reinforcing the idea that their benefit extends beyond metabolic regulation and further supports the argument that GLP-1RAs could be a widely accessible and scalable intervention, regardless of weight status.

Taken together, the findings from Farokhnia et al. (4) point to an exciting opportunity to redefine how we approach AUD pharmacotherapy. Rather than exclusively supporting abstinence-based treatment models, GLP-1RAs could play a transformative role in harm reduction, offering a medication that passively reduces alcohol consumption in individuals who might not otherwise engage with or have ready access to traditional AUD treatment services. This paradigm shift could have major public health implications, addressing the massive treatment gap in AUD by providing an option for those who do not necessarily identify as having an AUD but still engage in hazardous alcohol use. In a landscape where treatment accessibility and engagement remain substantial barriers, GLP-1RAs may offer a scalable solution to reducing alcohol-related harm at the population level.

As research continues, the challenge will be in determining how best to integrate GLP-1RAs into clinical practice. Should they be prescribed as a general harm-reduction tool for individuals with severe AUD? Could they be used as an early intervention strategy for those at risk of developing severe AUD? And how do we navigate the ethical and clinical implications of prescribing a medication that reduces alcohol use without requiring behavioral change? While these questions remain unanswered, one thing is clear: GLP-1RAs challenge the conventional approach to AUD treatment, offering an avenue that aligns more closely with real-world drinking behaviors and patient needs.

If we are willing to step beyond traditional abstinence-based paradigms, GLP-1RAs may pave the way for a more inclusive, effective, and pragmatic approach to reducing alcohol-related harm.

## Version 1 05/01/2025

Electronic publication

## References

[1] Drucker DJ (2024). The benefits of GLP-1 drugs beyond obesity. Science.

[2] Klausen MK (2022). Exenatide once weekly for alcohol use disorder investigated in a randomized, placebo-controlled clinical trial. JCI Insight.

[3] Hendershot CS Once-weekly semaglutide in adults with alcohol use disorder: a randomized clinical trial. JAMA Psychiatry.

[4] Farokhnia M (2025). Glucagon-like peptide-1 receptor agonists, but not dipeptidyl peptidase-4 inhibitors, reduce alcohol intake. J Clin Invest.

[5] Marty VN (2020). Long-acting glucagon-like peptide-1 receptor agonists suppress voluntary alcohol intake in male Wistar rats. Front Neurosci.

[6] Maisel NC (2013). Meta-analysis of naltrexone and acamprosate for treating alcohol use disorders: when are these medications most helpful?. Addiction.

